# New Imaging Method of Mobile Phone-Based Colorimetric Sensor for Iron Quantification

**DOI:** 10.3390/s25154693

**Published:** 2025-07-29

**Authors:** Ngan Anh Nguyen, Asher Hendricks, Emily Montoya, Amber Mayers, Diwitha Rajmohan, Aoife Morrin, Margaret McCaul, Nicholas Dunne, Noel O’Connor, Andreas Spanias, Gregory Raupp, Erica Forzani

**Affiliations:** 1 School of Engineering for Matter, Transport and Energy, Arizona State University, Tempe, AZ 85281, USA; annguye6@asu.edu (N.A.N.); ajpete20@asu.edu (A.H.); ermonto1@asu.edu (E.M.); anmayers@asu.edu (A.M.); drajmoha@asu.edu (D.R.); raupp@asu.edu (G.R.); 2 Center for Bioelectronics and Biosensors, Biodesign Institute, Arizona State University, Tempe, AZ 85281, USA; 3 School of Chemical Sciences, Dublin City University, D09 V209 Dublin, Ireland; aoife.morrin@dcu.ie (A.M.); margaret.mccaul@dcu.ie (M.M.); 4 Advanced Manufacturing Research Centre (I-Form), School of Mechanical and Manufacturing Engineering, Dublin City University, D09 NA55 Dublin, Ireland; nicholas.dunne@dcu.ie; 5 Biodesign Europe, Dublin City University, D09 NA55 Dublin, Ireland; 6 School of Electronic Engineering, Dublin City University, D09 V209 Dublin, Ireland; noel.oconnor@insight-centre.org; 7 Insight Centre for Data Analytics, Dublin City University, D09 V209 Dublin, Ireland; 8 School of Electrical, Computer and Energy Engineering, Arizona State University, Tempe, AZ 85281, USA; spanias@asu.edu; 9 Mayo Clinic, Scottsdale, AZ 85289, USA

**Keywords:** biosensors, image color analysis, iron detection, chemical sensors, smartphone-based detection, vertical flow assay, point-of-care sensor

## Abstract

Blood iron levels are related to many health conditions, affecting hundreds of millions of individuals worldwide. To aid in the prevention and treatment of iron-related disorders, previous research has developed a low-cost, accurate, point-of-care method for measuring iron from a single finger-prick blood sample. This study builds upon that work by introducing an improved imaging method that accurately reads sensor images irrespective of variations in environmental illumination and camera quality. Smartphone cameras were used as analytical tools, demonstrating an average coefficient of variation of 5.13% across different phone models, and absorbance results were found to be improved by 8.80% compared to the method in a previous study. The proposed method successfully enhances iron detection accuracy under diverse lighting conditions, paving the way for smartphone-based sensing of other colorimetric reactions involving various analytes.

## 1. Introduction

Iron metabolism plays a crucial role in maintaining human health [1]. Abnormal iron levels can lead to severe complications, including brain and liver damage [2]. Iron deficiency, the leading nutritional disorder globally, results from a lack of or dysfunctional red blood cells, ultimately causing anemia [3]. On the other hand, iron overload can lead to hemochromatosis, where excessive iron accumulates in body tissues [4].

Given that 40% of children and 30% of reproductive-age women suffer from anemia, while approximately 16 million Americans experience iron overload [5,6], a cost-effective and rapid iron monitoring method is essential. Prior research has introduced a point-of-care sensor capable of measuring iron biomarkers from whole blood via colorimetric chemistry [7,8]. However, existing methods require a specific phone model and controlled sensing conditions, such as a consistent light intensity, capturing angle, and distance. These limitations are not unique to iron sensing and are commonly encountered in other smartphone-based colorimetric detection platforms.

Color comparison has long served as the foundation of visual analytical techniques. Early approaches such as the Duboscq colorimeter, developed in the mid-19th century, allowed users to adjust optical path lengths to visually match the tint of an unknown solution to a reference standard [9]. Similarly, Nessler cylinders used paired glass tubes to facilitate concentration estimation through direct visual comparison under controlled lighting [9]. These classical methods, while effective in controlled laboratory settings, relied heavily on human judgment and required controlled environments and precise instrumentation.

Recent advancements in smartphone-based colorimetric detection have significantly enhanced point-of-care diagnostics and environmental monitoring. Balbach et al. developed Colourine, a smartphone app for urinalysis test strips that converts RGB data to HSV to reduce lighting interference; however, pre-calibration is required to establish baseline colors under specific lighting conditions [10]. Krishnan et al. introduced Krometriks, a smartphone-based accessory for colorimetric microRNA (miRNA) detection that achieved nanomolar sensitivity and laboratory-comparable performance across different phone models [11]. However, calibration with laboratory spectrophotometers was required for each smartphone to ensure accuracy [11]. In another approach, Mutlu et al. demonstrated that a smartphone camera alone can reliably classify pH levels from test strips using a least-squares support vector machine (LS-SVM) model [12]. Their method achieved high classification accuracy under various lighting conditions, but required extensive training with multiple pH values and lighting scenarios to build a robust model [12].

Despite these innovations, existing methods still require pre-calibration to address lighting variability and device-specific differences. Building on previous work in iron sensing and inspired by the long-standing principles of comparative colorimetry, as exemplified by the Duboscq colorimeter and Nessler cylinders, this study presents a modern adaptation of this classical concept. Specifically, it introduces an approach that embeds reference color cells directly within a disposable sensor, enabling in-image digital correction using smartphone-acquired RGB data. Unlike the visual and analog methods of the past, this design allows for consistent, quantitative colorimetric detection across various smartphone models and environmental conditions without the need for pre-calibration.

## 2. Materials and Methods

### 2.1. Sensor Fabrication

**Figure 1:**
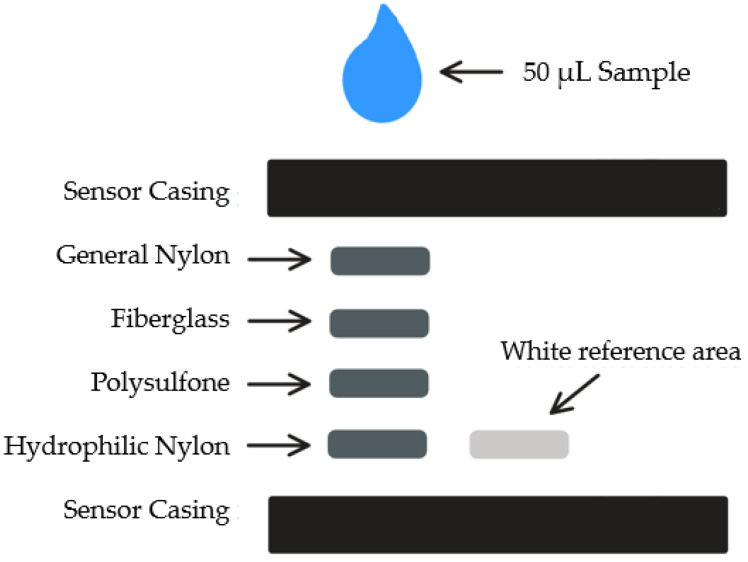
Schematic representation of the sensor design, illustrating the top three membrane layers responsible for sample preconditioning (e.g., separation of cells from plasma or particles from liquid) and the bottom membrane dedicated to colorimetric sensing via embedded chemical reagents.

The proposed design of the iron sensor consists of four different membrane layers designed for blood separation, with the fourth layer impregnated with capturing reagents for the iron-based chemical reaction. The four membranes used for the sensors in a previous publication include the following: general nylon membrane, fiberglass membrane, asymmetric polysulfone membrane, and hydrophilic nylon membrane [7]. On the side of the sensing area is the reference area which comprises white blotting paper, ensuring a stable reference for color analysis. The design of the sensor strip is illustrated in Figure 1.

The top and bottom sensor frames were 3D printed using an Ultimaker3 3D printer and the membrane layers were laser cut using Universal Laser Systems laser cutter in 6 by 6 mm squares [7]. All membranes were assembled between the top and bottom sensor frames and the ready-to-run sensors were individually packed and sealed with desiccant in aluminized Mylar bags. Calibration was conducted using a series of iron standards prepared from iron (III) nitrate nonahydrate (INN) crystals. All samples underwent spectrophotometric analysis in triplicate where the apparatus was in absorbance mode with an endpoint analysis of 590 nm [7,8].

According to a previous publication, the proposed colorimetric reaction chemistry involves two reagents: Reagent A, composed of 200 mM citric acid, 34 mM ascorbic acid, and 100 mM thiourea; and Reagent B, containing 6 mM ferene. These reagents are mixed with the iron-containing sample in a final volume ratio of 3:1:1 (Reagent A/Reagent B/Sample). Various reagent ratios and base chemical concentrations were evaluated in the previous study, and the selected 3:1:1 ratio was found to yield the most sensitive, consistent, and accurate results [8].

### 2.2. Sensor Testing

For use, the sensor is oriented so the sensor reading side is placed face down on a flat surface, then the liquid sample is inserted into the sampling port on the opposite side. After 10 min, the sensor is flipped with the sensor reading side face up and an image is captured using a smartphone for subsequent colorimetric analysis. Image acquisition is followed by RGB analysis, which is performed using ImageJ (version 1.54g, National Institutes of Health, Bethesda, MD, USA), an image processing software widely used in scientific research. The absorbance signals from red, green, and blue (RGB) components are calculated by the following equation:

(1) $$Absolute\;absorbance=-log\left(\frac{I}{I_{0}}\right)$$

where I is the intensity derived from the RGB deconvolution of the region of interest, and $I_{0}$ is the intensity of the white reference area within the same sensor.

### 2.3. Sensor Reading Correction Method

**Figure 2:**
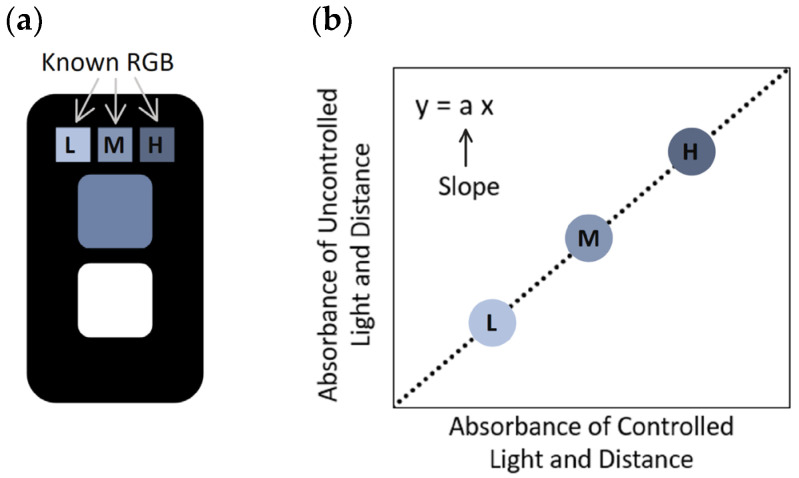
(**a**). Top view of sensor with three reference cells; (**b**). Illustration of the correlation plot of the uncontrolled vs. controlled absorbance from three reference cells.

As described in the previous work, a key limitation was that the white reference area performed poorly under varying lighting conditions, necessitating the use of a dedicated detection unit for accurate sensor signal assessment. To address this issue, the present study incorporated a three-reference-cell system into the sensor design, consisting of low-, medium-, and high-blue-intensity reference cells (as shown in Figure 2a).

The RGB values from the three blue reference cells, acquired under both controlled and uncontrolled lighting conditions, are used to compute a correction factor for each sensor image. Specifically, the RGB values are first converted to absorbance values using Equation (1). For each iron concentration, the average absorbance values of the reference cells under uncontrolled conditions are plotted against those from controlled conditions to generate a linear correlation plot (Figure 2b). The slope of this correlation (which quantifies lighting deviation) is then used to normalize the absorbance of the sensing area. This corrected absorbance value can be found by the following equation:

(2) $$Corrected\;abs=\frac{{Abs}_{Sensing\;}}{{Correlation\;Slope\;Abs}_{Blue\;Ref}}$$

where *Abs_Sensing_* is the absorbance value of the sensing area of the sensor (under uncontrolled conditions) and “*Correlation Slope Abs_Blue Ref_*” is the slope of the plot of uncontrolled lighting values against the previously mentioned controls. The corrected absorbance values were then compared to the gold-standard laboratory spectrophotometric method for accuracy analysis.

Other color palettes including red, gray, and green were also evaluated as reference cells (results provided in Supplementary Materials, Figure S1). However, after comparing different colors for use in these reference cells, it was found that varying intensities of blue exhibited the lowest coefficient of variation and thus gave the most accurate and consistent readings.

### 2.4. Phone Models Used for Sensor Imaging

The smartphone models used in this study were as follows: 1—iPhone XR (Apple Inc., Cupertino, CA, USA), 2—Samsung Galaxy S10+ (Samsung Electronics Co., Ltd., Suwon, South Korea), and 3—Samsung Note 8 (Samsung Electronics Co., Ltd., Suwon, South Korea). The Samsung Galaxy S10+ was used to capture images under controlled lighting conditions within a light box (light intensity: 1316 ± 3 lux), while all three phones were employed to capture sensor images under variable lighting conditions in different areas of the laboratory: on top of the lab bench (92 ± 2 lux), underneath the lab bench (6 ± 1 lux), and next to a window (1693 ± 3 lux).

To minimize the influence of automatic image processing such as filtering and color enhancement, all smartphone cameras were operated in manual mode with these enhancements disabled. Specifically, the Samsung Galaxy S10+ and Note 8 were configured in “Pro Mode” with RAW image capture enabled to provide unprocessed image data suitable for quantitative analysis. For the iPhone XR, the Halide Mark II camera application (Lux Optics Incorporated, San Francisco, CA, USA) was used to enable RAW capture and manual control of camera settings.

## 3. Results and Discussion

**Figure 3:**
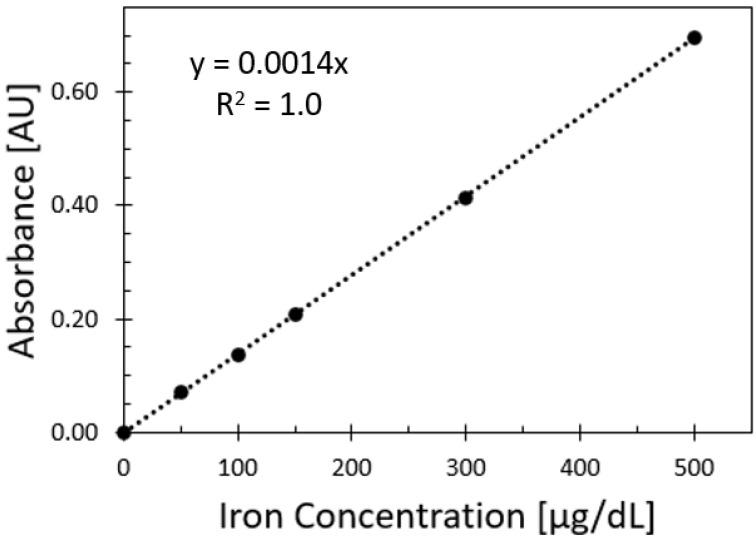
Spectrophotometric calibration curve for iron (III) nitrate nonahydrate (INN) at concentrations of 0, 50, 100, 150, 300, and 500 μg/dL. The coefficients of variation for these concentrations were 1.4%, 2.9%, 1.0%, 1.8%, 1.0%, 2.9%, and 1.4%, respectively. Error bars are included but not visible due to their minimal size.

Figure 3 shows the relationship between absorbance values and iron values from the spectrophotometer reference method. The measurements were obtained at 590 nm, and each sample was replicated three times for each iron concentration.

**Figure 4:**
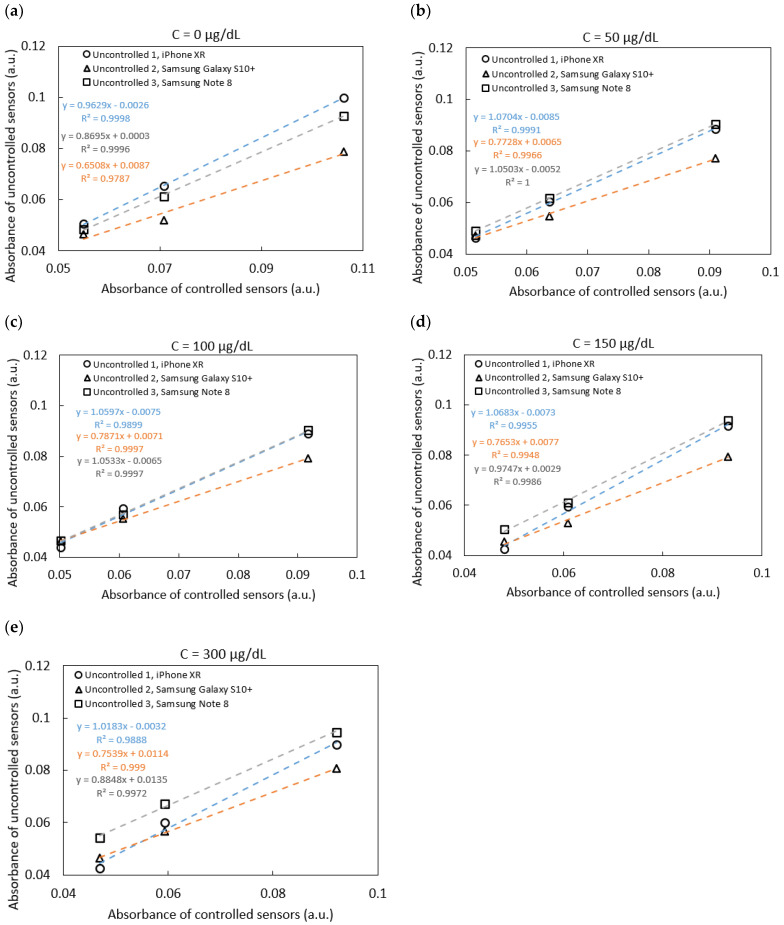
Absorbance correlation plots between sensors under uncontrolled and controlled conditions for iron concentrations: (**a**) 0 μg/dL; (**b**) 50 μg/dL; (**c**) 100 μg/dL; (**d**) 150 μg/dL; and (**e**) 300 μg/dL. Each data point represents the mean absorbance calculated from triplicate RGB measurements, with standard deviation < 3%.

**Figure 5:**
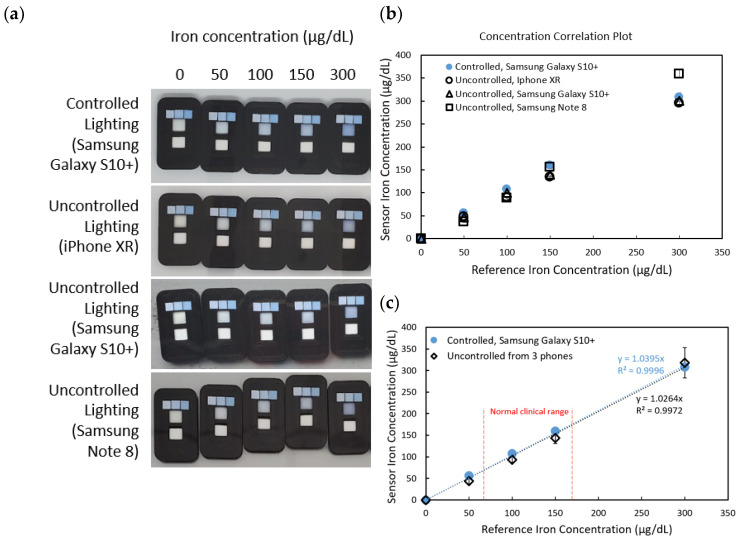
(**a**) Sensor images taken under controlled and uncontrolled lighting conditions using iPhone XR, Samsung Galaxy S10+, and Samsung Note 8 cameras, respectively. Left to right iron concentrations: 0, 50, 100, 150, and 300 μg/dL. (**b**) Concentration correlation plot of the sensor against the reference iron concentrations (spectrophotometry results); each data point represents the mean concentration calculated from triplicate RGB measurements of images across the three phone models. (**c**) Concentration correlation plot of the sensor against the reference iron concentrations. Uncontrolled values were obtained by averaging the sensor concentrations derived from the corrected data of the three phone models. The normal clinical range of iron levels found in human blood is also displayed.

Five sensors were tested using iron standards at concentrations of 0, 50, 100, 150, and 300 μg/dL. As shown in Figure 4a–e, the absorbance correlation plots illustrate varying slopes, which reflect differences in ambient lighting conditions during image capture. These slopes were used in Equation (2) to correct for lighting variability and obtain normalized absorbance values. The corrected values were subsequently compared to the known concentrations of the iron standards, as shown in Figure 5b,c. Intercept corrections were applied to account for systematic offsets observed between different phone models. After applying these corrections, the sensor readings across different phone models demonstrated strong agreement with the spectrophotometric reference, yielding an average coefficient of variation of 5.13%, indicating good reproducibility despite the variation in hardware and environmental lighting.

Although the absolute absorbance range obtained from RGB analysis was relatively narrow (approximately 0.04–0.1 a.u. across the tested concentration range, as shown in Figure 4), the system demonstrated high signal-to-noise ratio (SNR), enabling consistent quantification. The minimal baseline noise (approximately 10^−4^ a.u.) and low variability in triplicate RGB measurements (CV < 3%) contributed to a high SNR even at low iron concentrations. This high SNR is critical for ensuring analytical sensitivity, especially when the absolute signal change is subtle. It enables the system to distinguish small but reproducible differences in absorbance, which can be reliably translated into iron concentration estimates.

Figure 5a showcases sensor images captured under both controlled and uncontrolled lighting conditions using three different smartphone models (iPhone XR, Samsung Galaxy S10+, and Samsung Note 8). The pictures indicate significant variations in color perception due to environmental lighting and device-specific image processing. However, applying the correction method using the three-reference-cell system significantly improves the accuracy of iron concentration readings.

As seen in Figure 5b,c, the concentrations suggested from the sensor correlate well with the spectrophotometer data. Sensor measurements for the normal clinical blood iron range (60–170 μg/dL) display a good match of the uncontrolled compared to the controlled sensors. With the absorbance correction method, the results were found to be improved by 8.80% compared to the old method that did not apply the three-reference-cell correction system.

**Figure 6:**
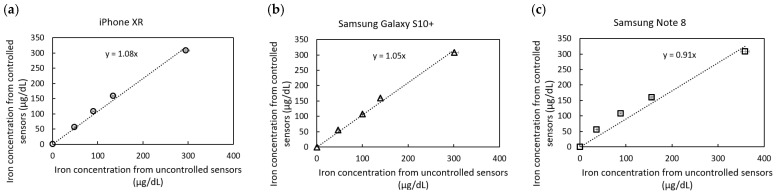
Iron concentration correlation plot between controlled and uncontrolled lighting conditions using (**a**) iPhone XR; (**b**) Samsung Galaxy S10+; and (**c**) Samsung Note 8 cameras. Each data point represents the mean concentration calculated from triplicate RGB measurements.

Figure 6 presents correlation plots of predicted iron concentrations under controlled and uncontrolled lighting conditions for three different smartphone models. The iPhone XR and Samsung Galaxy S10+ slightly overestimated iron concentrations by 8% and 5%, respectively, while the Samsung Note 8 underestimated concentrations by 9%. Despite these variations, the proposed correction method effectively normalizes the results, bringing them within an acceptable margin of error (±10% as user reference).

These findings underscore the robustness of the correction method, demonstrating its ability to standardize measurements across different phone models and lighting conditions. The primary objective of this study was not to evaluate the performance of the latest smartphone models, but rather to validate a device-independent image correction method capable of functioning across diverse devices. This approach prioritizes cross-device adaptability, which is critical for practical deployment in real-world, low-resource settings.

The improved accuracy of iron detection using a smartphone-based system presents significant implications for point-of-care diagnostics, particularly in low-resource settings where laboratory spectrophotometers may not be readily available. While this study employed widely available smartphone models (iPhone XR, Samsung Galaxy S10+, and Samsung Note 8), ongoing work is evaluating the method’s performance using newer smartphone models to further validate robustness across a broader market segment.

## 4. Conclusions

This study successfully developed and validated an enhanced color detection method for smartphone-based colorimetric iron sensors. The incorporation of three reference cells for RGB color analysis significantly improved the reliability of the sensor readings, making them less dependent on environmental lighting conditions and phone model variations. This approach holds promise for future applications in colorimetric sensing for various analytes, broadening the potential of smartphone-based diagnostic tools.

## Data Availability

Data is contained within the article.

## Supplementary Materials

The following supporting information can be downloaded at https://www.mdpi.com/article/10.3390/s25154693/s1: Figure S1: (a,c,e) Sensor images taken under controlled lighting conditions using Samsung Galaxy S10+ camera with different reference cell color palettes: red, gray, and green, respectively. Left to right iron concentrations: 0, 50, 100, 150, and 300 μg/dL; (b,d,f) Concentration correlation plots of sensor against the reference iron concentrations (spectrophotometry results). Uncontrolled values were obtained using images captured under uncontrolled lighting with the same phone model (Samsung Galaxy S10+). Each data point represents the mean concentrations calculated from triplicate RGB measurements, with standard deviation < 5%.

## References

[1] Kaplan L.A., Pesce A.J., Kazmierczak S.C. (2003). Clinical Chemistry: Theory, Analysis, Correlation.

[2] Beutler E., Hoffbrand A.V., Cook J.D. (2003). Iron deficiency and overload. Hematology.

[3] Stevens G.A., Finucane M.M., De-Regil L.M., Paciorek C.J., Flaxman S.R., Branca F., Peña-Rosas J.P., Bhutta Z.A., Ezzati M. (2013). Global, regional, and national trends in haemoglobin concentration and prevalence of total and severe anaemia in children and pregnant and non-pregnant women for 1995–2011: A systematic analysis of population-representative data. Lancet Glob. Health.

[4] Pietrangelo A. (2016). Iron and the liver. Liver Int..

[5] Safiri S., Kolahi A.-A., Noori M., Nejadghaderi S.A., Karamzad N., Bragazzi N.L., Sullman M.J.M., Abdollahi M., Collins G.S., Kaufman J.S. (2021). Burden of anemia and its underlying causes in 204 countries and territories, 1990–2019: Results from the Global Burden of Disease Study 2019. J. Hematol. Oncol..

[6] McDowell L.A., Kudaravalli P., Sticco K.L. (2022). Iron Overload.

[7] Serhan M., Jackemeyer D., Abi Karam K., Chakravadhanula K., Sprowls M., Cay-Durgun P., Forzani E. (2021). A novel vertical flow assay for point of care measurement of iron from whole blood. Analyst.

[8] Serhan M., Jackemeyer D., Long M., Sprowls M., Perez I.D., Maret W., Chen F., Tao N., Forzani E. (2020). Total iron measurement in human serum with a novel smartphone-based assay. IEEE J. Transl. Eng. Health Med..

[9] Stock J.T. (1994). The Duboscq Colorimeter and Its Inventor. J. Chem. Educ..

[10] Balbach S., Jiang N., Moreddu R., Dong X., Kurz W., Wang C., Dong J., Yin Y., Butt H., Brischwein M. (2021). Smartphone-based colorimetric detection system for portable health tracking. Anal. Methods.

[11] Krishnan T., Wang H.-N., Vo-Dinh T. (2021). Smartphone-Based Device for Colorimetric Detection of MicroRNA Biomarkers Using Nanoparticle-Based Assay. Sensors.

[12] Mutlu A.Y., Kılıç V., Özdemir G.K., Bayram A., Horzum N., Solmaz M.E. (2017). Smartphone-based colorimetric detection via machine learning. Analyst.

