# Supplementary material for: New Imaging Method of Mobile Phone-Based Colorimetric Sensor for Iron Quantification
Source: Sensors (Basel). 2025 Jul 29;25(15):4693. doi: 10.3390/s25154693 (PMC12349305; doi:10.3390/s25154693)
Supplement: Supplementary file 1 [file sensors-25-04693-s001.zip › sensors-3687789-supplementary.pdf]

Supplementary Information

New Imaging Method of Mobile Phone-Based Colorimetric Sensor for Iron Quantification

Ngan Anh Nguyen [1,2], Asher Hendricks [1,2], Emily Montoya [1], Amber Mayers [1], Diwitha Rajmohan [1], Aoife Morrin [3], Margaret McCaul [3], Nicholas Dunne [4], Noel O'Connor [5,6], Andreas Spanias [7], Gregory Raupp [1], and Erica Forzani [1,2,8,*]

1 School of Engineering for Matter, Transport and Energy, Arizona State University, Tempe, AZ 85281, USA; annguye6@asu.edu (N.A.N.); ajpete20@asu.edu (A.H.); ermonto1@asu.edu (E.M.); anmayers@asu.edu (A.Ma.); drajmoha@asu.edu (D.R.); raupp@asu.edu (G.R.); eforzani@asu.edu (E.F.)
2 Center for Bioelectronics and Biosensors, Biodesign Institute, Arizona State University, Tempe, AZ 85281, USA
3 School of Chemical Sciences, Dublin City University, Dublin 9, Ireland; aoife.morrin@dcu.ie (A.Mo.); margaret.mccaul@dcu.ie (M.M.)
4 School of Mechanical and Manufacturing, Dublin City University, Dublin 9, Ireland; nicholas.dunne@dcu.ie (N.D.)
5 School of Electronic Engineering, Dublin City University, Dublin 9, Ireland; noel.oconnor@insight-centre.org (N.O.)
6 Insight Centre for Data Analytics, Dublin City University, Dublin 9, Ireland
7 School of Electrical, Computer and Energy Engineering, Arizona State University, Tempe, AZ 85281, USA; spanias@asu.edu (A.S.)
8 Mayo Clinic, Scottsdale, AZ 85289, USA
* Correspondence: eforzani@asu.edu

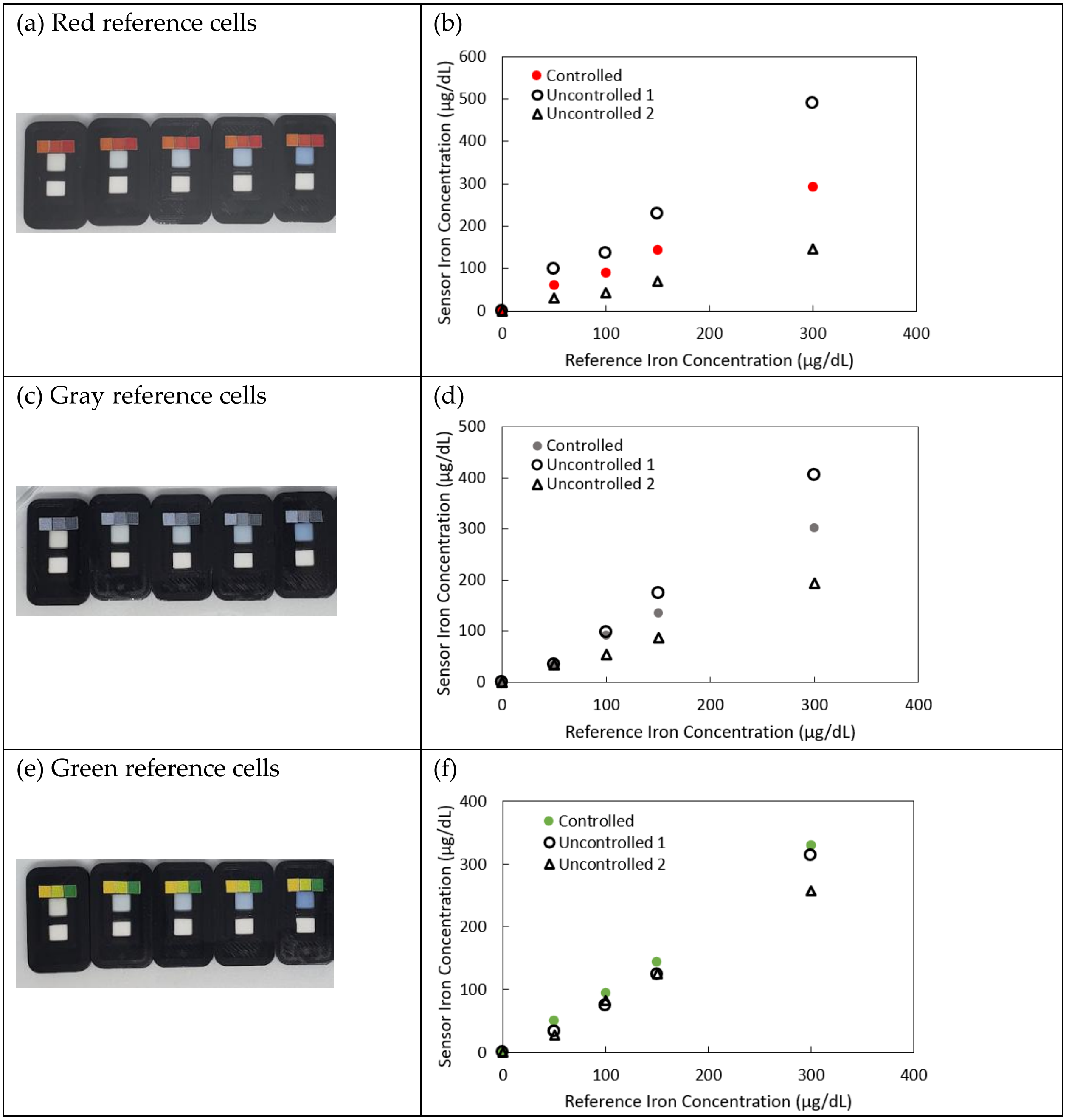

Figure S1. (**a, c, e**) Sensor images taken under controlled lighting conditions using Samsung Galaxy S10+ camera with different reference cell color palettes: red, gray, and green, respectively. Left to right iron concentrations: 0, 50, 100, 150, and 300 µg/dL; (**b, d, f**) Concentration correlation plots of sensor against the reference iron concentrations (spectrophotometry results). Uncontrolled values were obtained using images captured under uncontrolled lighting with the same phone model (Samsung Galaxy S10+). Each data point represents the mean of concentrations calculated from triplicate RGB measurements, with standard deviation < 5%.
